# Protease‐activated receptor‐1 contributes to renal injury and interstitial fibrosis during chronic obstructive nephropathy

**DOI:** 10.1111/jcmm.14028

**Published:** 2018-11-28

**Authors:** Maaike Waasdorp, Dennis M. de Rooij, Sandrine Florquin, JanWillem Duitman, C. Arnold Spek

**Affiliations:** ^1^ Center for Experimental and Molecular Medicine, Academic Medical Center Amsterdam Amsterdam The Netherlands; ^2^ Pathology Academic Medical Center Amsterdam Amsterdam The Netherlands; ^3^ Département Hospitalo‐Universitaire FIRE (Fibrosis, Inflammation and Remodeling) and LabEx Inflamex Université Paris Diderot, Sorbonne Paris Cité Paris France; ^4^ Physiopathologie Et Epidémiologie Des Maladies Respiratoires, Medical School Xavier Bichat, Inserm UMR1152 Paris France

**Keywords:** epithelial‐to‐mesenchymal transition, obstructive nephropathy, protease‐activated receptor‐1, renal fibrosis, unilateral ureter obstruction

## Abstract

End‐stage renal disease, the final stage of all chronic kidney disorders, is associated with renal fibrosis and inevitably leads to renal failure and death. Transition of tubular epithelial cells (TECs) into mesenchymal fibroblasts constitutes a proposed mechanism underlying the progression of renal fibrosis and here we assessed whether protease‐activated receptor (PAR)‐1, which recently emerged as an inducer of epithelial‐to‐mesenchymal transition (EMT), aggravates renal fibrosis. We show that PAR‐1 activation on TECs reduces the expression of epithelial markers and simultaneously induces mesenchymal marker expression reminiscent of EMT. We next show that kidney damage was reduced in PAR‐1‐deficient mice during unilateral ureter obstruction (UUO) and that PAR‐1‐deficient mice develop a diminished fibrotic response. Importantly, however, we did hardly observe any signs of mesenchymal transition in both wild‐type and PAR‐1‐deficient mice suggesting that diminished fibrosis in PAR‐1‐deficient mice is not due to reduced EMT. Instead, the accumulation of macrophages and fibroblasts was significantly reduced in PAR‐1‐deficient animals which were accompanied by diminished production of MCP‐1 and TGF‐β. Overall, we thus show that PAR‐1 drives EMT of TECs in vitro and aggravates UUO‐induced renal fibrosis although this is likely due to PAR‐1‐dependent pro‐fibrotic cytokine production rather than EMT.

## 1 INTRODUCTION

End‐stage renal disease (ESRD) is considered to be the final stage of chronic kidney disease, independent of the underlying cause.^1^ ESRD is associated with renal fibrosis and, apart from blood pressure control to slow its progression, there are no specific treatments to prevent or resolve renal fibrosis. In patients diagnosed with ESRD, renal replacement therapy, either transplantation or dialysis, are the only treatment options to date. As both options are a huge burden to patients, alternative (preventive) treatment options are eagerly awaited for. Consequently, better insight into the molecular pathogenesis of renal fibrosis is warranted.

Epithelial to mesenchymal transition (EMT) of tubular epithelial cells (TECs), a phenotypic conversion programme characterized by the loss of epithelial markers and gain of mesenchymal features, is considered one of the mechanisms contributing to the onset and pathogenesis of renal fibrosis. After injury, TECs undergo EMT in order to avoid imminent cell death and to aid tissue repair.^2^, ^3^, ^4^, ^5^ Dysregulated repair due to persistent injury, however, leads to a switch from a regenerative process into a detrimental fibrotic response.^6^ Although the concept of TECs undergoing mesenchymal transition upon kidney damage was raised more than a decade ago, the molecular mechanisms that control EMT of TECs remain largely unidentified.

Interestingly, the family of protease‐activated receptors (PARs) recently emerged as key players in EMT.^7^ PARs are G‐protein coupled receptors that are activated by coagulation proteases thereby enabling these proteases to influence a range of pathophysiologic processes.^8^ As opposed to classical G‐protein coupled receptors, PAR activation requires proteolytic cleavage rather than ligand binding. Indeed, after proteolytic removal of the N‐terminal extracellular region, a novel tethered ligand that interacts with the body of the receptor is unmasked. Subsequent transmembrane signalling leads, amongst others, to EMT of alveolar epithelial cells and retinal pigment epithelial cells.^9^, ^10^, ^11^ Moreover, PAR‐1‐dependent signalling drives fibroblast proliferation and extracellular matrix production in vitro, whereas PAR‐1 deficiency limits liver, lung and skin fibrosis in experimental animal models.^12^, ^13^, ^14^, ^15^ In the kidney, PAR‐1 is expressed by endothelial cells, podocytes, mesangial cells, and tubular epithelial cells^16^ and we recently showed that PAR‐1 potentiates diabetic nephropathy by inducing mesangial cell proliferation and extracellular matrix production.^17^

Based on the key role of PAR‐1 in fibroproliferative disease, it is thus tempting to speculate that PAR‐1 may be a key factor driving the pathogenesis of renal fibrosis. We challenged this hypothesis by evaluating renal fibrosis in wild‐type and PAR‐1‐deficient mice subjected to the well‐established murine unilateral ureter obstruction (UUO) model.

## 2 MATERIALS AND METHODS

### 2.1 Mice

PAR‐1‐deficient mice, generated on a C57Bl/6 background, were purchased from The Jackson Laboratory (Bar Harbor, ME, USA) whereas wild‐type C57BL/6 mice were purchased from Charles River (Maastricht, the Netherlands). All experiments were approved by the Institutional Animal Care and Use Committee of the University of Amsterdam and maintained according to institutional guidelines. Animal procedures were carried out in compliance with the Institutional Standards for Humane Care and Use of Laboratory Animals of the Academic Medical Center. The Animal Care and Use Committee of the Academic Medical Center approved all experiments.

### 2.2 Unilateral Ureter Obstruction Model

Thirty‐two wild‐type and 32 PAR‐1‐deficient C57Bl/6 mice were subjected to the UUO model as described before.^18^, ^19^ Briefly, 8‐week‐old mice received preoperative analgesia (subcutaneous injection of 100 μg/kg buprenorphine (Temgesic, Shering‐Plough)) and the right ureter was subsequently double ligated with 6.0 silk through a small abdominal incision under 2.0% isoflurane‐induced anaesthesia. Six wild‐type and six PAR‐1‐deficient mice received a sham operation, in which all procedures were followed apart from ligation of the ureter. Eight mice of each genotype were killed either 1, 3, 7, or 10 days after surgery. Blood and kidneys were harvested and prepared for further analysis. Each kidney was divided in halves; one half was fixed in 4% formalin and embedded in paraffin and the other half was homogenized for protein and RNA analysis. Contralateral non‐obstructed kidneys served as control.

### 2.3 Cell culture and stimulation

Conditionally immortalized proximal tubular epithelial cells (imPTECs^20^) were cultured according to routine procedures using a 1:1 mixture of Dulbecco's modified Eagle's medium containing 1 g/L glucose with Ham's‐F12 medium, supplemented with heat inactivated calf serum (10%), 100 U/mL penicillin, 100 μg/mL streptomycin, 2 mM L‐glutamine, 5 μg/mL insulin, 5 μg/mL transferrin, 5 μg/mL selenite, 20 ng/mL Tri‐iodo‐thyrionine, 50 ng/mL Hydrocortisone, and 5 ng/mL Prostaglandin E1. Cells were cultured at 33°C in 5% CO_2_% and 95% air. One week prior to experiments, cells were washed in PBS and cultured at 37°C to allow SV40 down‐regulation and consequent differentiation. Prior to each experiment, imPTEC differentiation was determined based on morphology. Cells were starved at least 4 hours prior to stimulation with 1 U/mL thrombin (Sigma, St. Louis, Missouri, USA), 100 μM PAR‐1 agonist peptide (TRAP6; H‐SFLLRN‐NH2; Biochem, Shanghai, China), or 5 μg/mL TGF‐β (Tebu‐bio, Heerhugowaard, the Netherlands).

### 2.4 RNA isolation and RT qPCR

For gene expression analysis, mRNA was isolated from kidney homogenates or cultured cells using TriReagent (#11667165001; Roche Diagnostics) according to the manufacturer's recommendations. All mRNA samples were quantified by spectrophotometry and stored at −80°C until further analysis. One microgram of mRNA was treated with RQ1 DNAse (M6101, Promega, Madison, WI, USA) and subsequently converted to cDNA using M‐MLV reverse transcriptase (M1705, Promega, Madison, WI, USA) and random hexamer primers (#SO142, Fisher scientific, Landsmeer, the Netherlands) according to the manufacturer's recommendations. qPCR and subsequent analysis were performed using sensiFAST No‐ROX PCR master mix (GC Biotech) on a Lightcycler 480 machine and corresponding software (Software release 1.5.0 (1.5.0.39), Roche, Almere, the Netherlands). Expression levels were normalized using the average expression levels of β‐actin, GAPDH and TBP. Primer sequences are listed in Table 1.

**Table 1:** Primer sequences used for quantitative PCR analysis

Gene	Full name	Forward primer (5′‐3′)	Reverse primer (5′‐3′)
*F2r*	Protease‐activated receptor 1	GTTGATCGTTTCCACGGTCT	ACGCAGAGGAGGTAAGCAAA
*Aqp1*	Aquaporin 1	AGGCTTCAATTACCCACTGGA	GTGAGCACCGCTGATGTGA
*Vim*	Vimentin	GCTGCGAGAGAAATTGCAGGA	CCACTTTCCGTTCAAGGTCAAG
*Acta2*	smooth muscle‐α‐actin	TCCCTGGAGAAGAGCTACGAACT	GATGCCCGCTGACTCCAT
*Tjp1*	Zona Occludens 1	GCCGCTAAGAGCACAGCAA	GCCCTCCTTTTAACACATCAGA
*Fn1*	Fibronectin	ATGTGGACCCCTCCTGATAGT	GCCCAGTGATTTCAGCAAAGG
*Col1a1*	Collagen I	GCTCCTCTTAGGGGCCACT	CCACGTCTCACCATTGGGG
*Adgre1*	F4/80	CTTTGGCTATGGGCTTCCAGTC	GCAAGGAGGACAGAGTTTATCGTG
*Tnf*	Tumor necrosis factor α	CTGTAGCCCACGTCGTAGC	TTGAGATCCATGCCGTTG
*Ccl2*	Macrophage chemoattractant protein 1	CATCCACGTGTTGGCTCA	GATCATCTTGCTGGTGAATGAGT
*IL1b*	Interleukin 1β	TGAGCACCTTCTTTTCCTTCA	TTGTCTAATGGGAACGTCACAC
*Il6*	Interleukin 6	GCTACCAAACTGGATATAATCAGGA	CCAGGTAGCTATGGTACTCCAGAA
*Tgfb1*	Transforming growth factor β1	CTGACCCCCACTGATACGCCT	TGCTGTCACAAGAGCAGTGAGC
*Tgfb3*	Transforming growth factor β3	CTGTTGAGGAGAGAGTCCAACTTG	CCAGTATGTCTCCATTGGGCTGA
*Cxcl1*	KC	ATAATGGGCTTTTACATTCTTTAACC	AGTCCTTTGAACGTCTCTGTCC
*Tbp*	Tata‐binding protein	CCTTGTACCCTTCACCAATGAC	ACAGCCAAGATTCACGGTAGA
*Gapdh*	Glyceraldehyde 3‐phosphate dehydrogenase	CTCATGACCACAGTCCATGC	CACATTGGGGGTAGGAACAC
*Actb*	β‐actin	GTGACGTTGACATCCGTAAAGA	GCCGGACTCATCGTACTCC

### 2.5 Western blot

Cells were seeded at a density of 20 000 cells/well in 24‐well plates. After stimulation for 24 hours, cells were washed in ice‐cold PBS and lysed in Laemmli buffer. Kidney homogenates were lysed in Greenberger Lysis buffer. Lysates were next separated on 8%‐12% SDS‐PAGE gels and transferred onto Immobulin‐P membranes (Millipore) as described before.^21^ Membranes were blocked for 1 hour at room temperature in 5% bovine serum albumin (BSA) in TBS+0.1% tween‐20 (TBS‐T) and subsequently incubated with the following primary antibodies, diluted in TBS‐T: mouse‐anti‐tubulin 1:1000 (Santa Cruz; sc‐23948); mouse‐anti‐GAPDH 1:1000 (Santa Cruz; sc‐32233); mouse‐anti‐b‐actin 1:1000 (Santa Cruz; sc‐81178); goat‐anti‐fibronectin 1:1000 (Santa Cruz; sc‐6953); mouse‐anti‐α‐SMA 1:1000 (Santa Cruz; sc‐32251); rabbit‐anti‐vimentin 1:1000 (Cell Signaling; #5741); rabbit‐anti‐ZO‐1 1:1000 (Thermo Fisher; 617300); mouse‐anti‐AQP‐1 1:1000 (Santa Cruz; sc‐25287); goat‐anti‐Collagen I 1:1000 (Southern Biotech; 1310‐01); rabbit‐anti‐fibronectin 1:1000 (Abcam; ab134184); rabbit‐anti‐SGLT2 1:1000 (BioVision; 3690‐100);. After overnight incubation, the membranes were washed with TBS‐T and incubated for 1 hour at room temperature with horseradish peroxidase (HRP)‐conjugated anti‐mouse‐IgG (Dako; P0447), anti‐goat‐IgG (Dako; P0160) or anti‐Rabbit‐IgG (Cell Signaling; #7074) diluted 1:5000 in TBS‐T. Membranes were washed in TBS and imaged using Lumi‐Light (12015200001; Roche, Basel, Switzerland) on an ImageQuant LAS 4000 biomolecular imager (GE Healthcare, Zeist, the Netherlands).

### 2.6 Protein simple Wes

e‐Cadherin protein levels were detected in kidney homogenate lysates using the Protein Simple Wes according to the manufacturer's instructions. Rabbit‐anti‐eCadherin 1:50 (Cell Signaling; #3195S) primary antibody, Anti‐Rabbit Detection Module kit (ProteinSimple; DM‐001) and 12‐230 kDa Wes Separation Module, 8 x 25 capillary cartridges (ProteinSimple;SM‐W004) were used.

### 2.7 Immunocytochemistry

Cells were seeded at a density of 20 000 cells/well on coverslips in 24‐well plates. After the indicated stimulation for 24‐72 hours, cells were washed in ice‐cold PBS, fixed in 4% paraformaldehyde in PBS and stained as described before.^22^ In short, cells were washed with PBS and incubated for 30 minutes in 0.1% Triton X‐100% and 0.5% BSA in PBS for blocking and permeabilization. Subsequently, cells were incubated with the following primary antibodies: rabbit‐anti‐ZO‐1 (1:200; Thermo Fisher; 617300) or mouse‐anti‐α‐SMA (1:1000; Santa Cruz; sc‐32251). After overnight incubation, cells were washed with PBS and incubated with Alexa488‐linked secondary anti‐mouse or anti‐rabbit antibodies for 1 hour. The cytoskeleton was stained using phalloidin (100 nM in PBS, 30 minutes) and nuclei were stained with DAPI (10 minutes). Coverslips were mounted onto object glass slides using prolong gold antifade reagent (Thermo Fisher). Micrographs were made using a Leica DM5000B fluorescent microscope with LAS X software (Leica).

### 2.8 (Immuno)histochemistry

Formalin‐fixed, paraffin‐embedded, 4‐μm‐thick kidney slides were stained with periodic acid–Schiff–diastase (PAS‐D) and picrosirius red following routine procedures. The PAS‐D slides were subsequently scored by a pathologist in a blinded fashion as previously described.^18^ Specific immunostainings were performed as described before^17^ using the following antibodies: rat‐anti‐F4/80 (1:500; clone CI:A3‐1, Serotec MCA497GA), rabbit‐anti‐vimentin (1:1000; #5741; Cell Signaling), mouse‐anti‐ α‐SMA (1:800; 1A4; sc‐32251; Santa Cruz Biotechnology). In short, paraffin‐embedded slides were deparaffinized, followed by endogenous peroxidase inhibition (15 minutes incubation in 0.3% H_2_O_2_ in methanol at room temperature). Slides were boiled in citrate buffer (pH6.0) for 10 minutes, blocked with normal goat serum or Ultra V block (Thermo Scientific, Runcorn, UK) for 30 minutes, and incubated with the primary antibody. After overnight incubation, slides were washed in PBS and incubated with rabbit‐anti‐rat IgG (1:3000) and HRP conjugated rabbit‐anti‐goat IgG (P0160; Dako) or goat‐anti‐mouse IgG2A (1:100) for 30 minutes at room temperature, visualized with DAB (BS04‐999; Immunologic) and counterstained using haematoxylin. Slides incubated without the primary antibody were used as negative controls to exclude non‐specific binding of the secondary antibody. Microphotographs were taken at 20 times magnification using a Leica DM5000B microscope equipped with a Leica DFC500 camera and Image Pro Plus software (vs 5.02; Media Cybernatics).

### 2.9 Statistics

All values are expressed as mean ± SEM. All groups were tested for normality using the D'Agostino‐Pearson omnibus normality test. Detected outliers were excluded from analysis. Differences between two groups were analysed using a *t* test if data were normally distributed, or a Mann‐Whitney *U* test for non‐parametric data. Multiple comparisons were analysed using one‐way‐ANOVA analysis or Kruskal‐Wallis test (for nonparametric values), followed by Bonferroni's or Dunns multiple comparison tests, respectively. All analyses were performed using GraphPad Prism version 5.01.

## 3 RESULTS

### 3.1 PAR‐1 activation induces EMT in tubular epithelial cells in vitro

**Figure 1:**
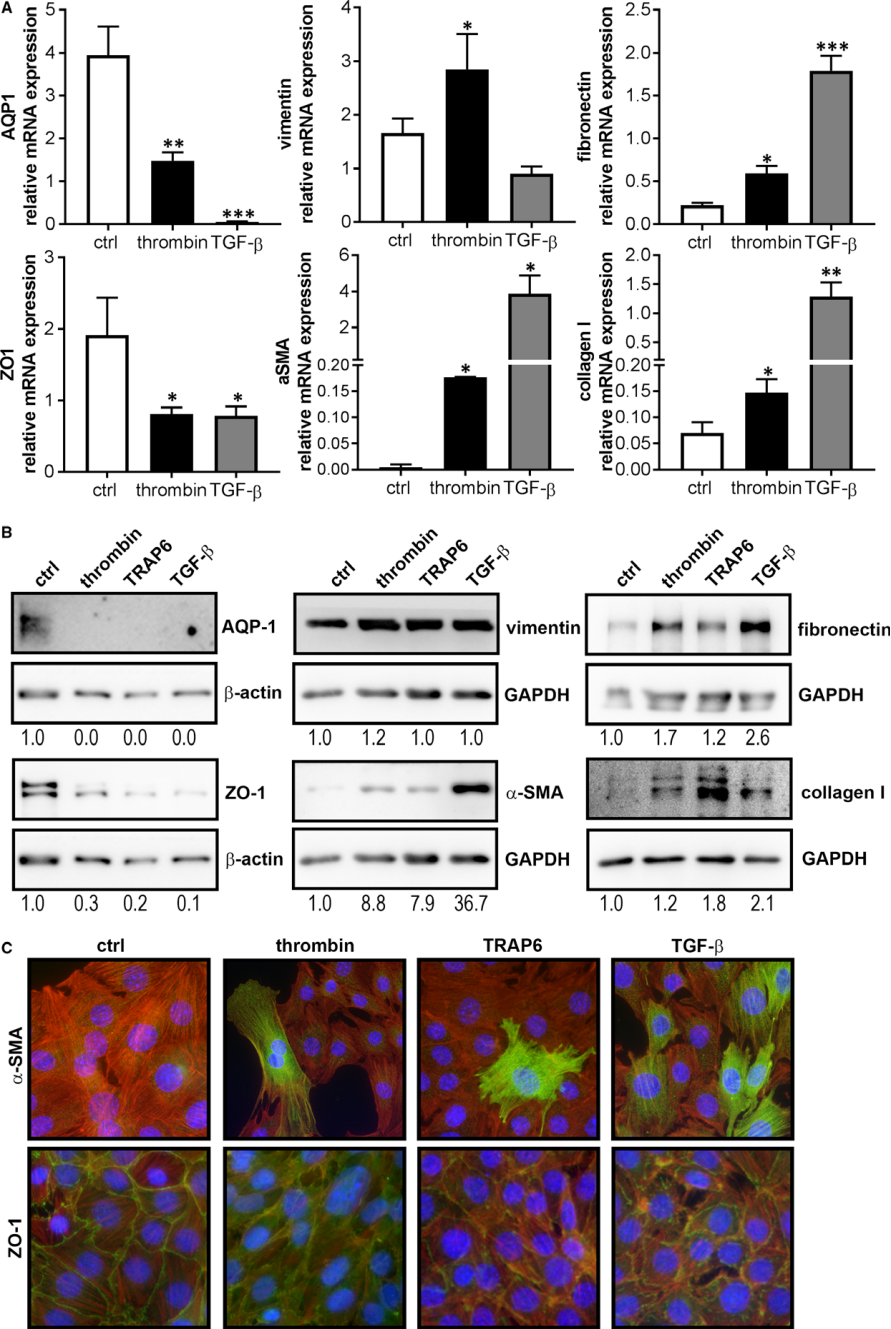
PAR‐1 activation induces mesenchymal transition of imPTECs. A, Relative mRNA expression levels of AQP‐1, ZO‐1, vimentin, α‐SMA, fibronectin, and collagen I in imPTECs 24 hours after stimulation with thrombin (1 U/mL) or TGF‐Β (5 ng/mL). Indicated is the average of three independent experiments. *P < 0.05, **P < 0.01, ***P < 0.005 (one‐way ANOVA followed by Bonferroni multiple comparisons test). B, Protein expression levels of AQP‐1, ZO‐1, vimentin, α‐SMA, fibronectin, and collagen I in imPTEC whole cell lysates 72 h after stimulation with thrombin (1 U/mL), TRAP‐6 (100 μM), or TGF‐Β (5 ng/mL). GAPDH or β‐actin expression served as a loading control. C: Representative images of imPTECs 72 h after stimulation with thrombin (1 U/ml), TRAP‐6 (100 μM), or TGF‐Β (0.5 ng/mL). Blue: DAPI, red: phalloidin, and green: α‐SMA (upper panels) and ZO‐1 (lower panels)

To test the hypothesis that PAR‐1 signalling induces EMT of TECs, immortalized murine proximal TECs (imPTECs) were stimulated with thrombin (prototypical PAR‐1 agonist), TRAP6 (specific PAR‐1 agonist peptide), or TGF‐β (well‐known inducer of tubular EMT serving as a positive control). As shown in Figure 1A, PAR‐1 stimulation induced EMT as evident from a shift to a mesenchymal gene expression profile with increased expression levels of key mesenchymal markers α‐SMA and vimentin, and decreased expression levels of tubular epithelial markers aquaporin‐1 (AQP1) and zona occludens‐1 (ZO1). Moreover, both PAR‐1 and TGF‐β stimulation induced mRNA expression of the extracellular matrix proteins collagen I and fibronectin. To substantiate these findings, we performed protein expression analysis by western blot and as shown in Figure 1B, PAR‐1 activation with thrombin or TRAP6 and stimulation with TGF‐β resulted in reduced AQP‐1 and ZO‐1 expression, increased α‐SMA expression, and production of collagen I and fibronectin. The fact that PAR‐1 activation induces EMT was confirmed by immunofluorescence. Indeed, as illustrated in Figure 1C, PAR‐1 activation led to an increase of α‐SMA expression, which was accompanied by diminished ZO‐1 expression and consequent disruption of the epithelial monolayer. Together, these results show that PAR‐1 stimulation leads to EMT of imPTECs in vitro.

### 3.2 PAR‐1 deficiency reduces UUO‐induced renal fibrosis in mice

**Figure 2:**
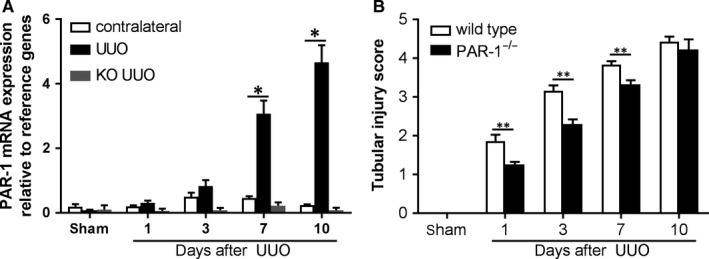
General evaluation of renal damage after UUO. A, PAR‐1 mRNA expression in kidney lysates of contralateral and obstructed (UUO) kidneys from wild‐type and PAR‐1‐deficient mice 1, 3, 7, and 10 d after UUO. B, Tubular injury score of PAS‐D‐stained kidney sections of mice killed at the indicated time points after the induction of UUO. *P < 0.05; **P < 0.01; (one‐way ANOVA followed by Bonferroni multiple comparisons test)

Based on our in vitro findings showing that PAR‐1 activation triggers EMT of TECs, we hypothesized that PAR‐1 would contribute to renal interstitial fibrosis. To prove or refute this hypothesis, PAR‐1‐deficient mice were subjected to UUO and fibrotic responses were compared to wild‐type controls. We first assessed PAR‐1 expression levels in control (contralateral) and obstructed kidney sections of wild‐type mice. As shown in Figure 2A, PAR‐1 expression is low in non‐obstructed kidneys and significantly increases 7 and 10 days after the induction of UUO (which is in line with a recent study showing PAR‐1 induction after UUO using immunohistochemistry^23^). As expected, no PAR‐1 mRNA was measured in the PAR‐1‐deficient mice during the experiment (Figure 2A). As shown in Figure 2B, PAR‐1‐deficient mice subjected to UUO developed less kidney injury based on assessment of PAS‐D stained kidney sections for tubular dilatation, brush border loss and tubular atrophy. Kidney damage was reduced in PAR‐1‐deficient mice as compared to wild types up to day 7 whereas ten days after UUO induction kidney damage was almost maximal in both genotypes.

**Figure 3:**
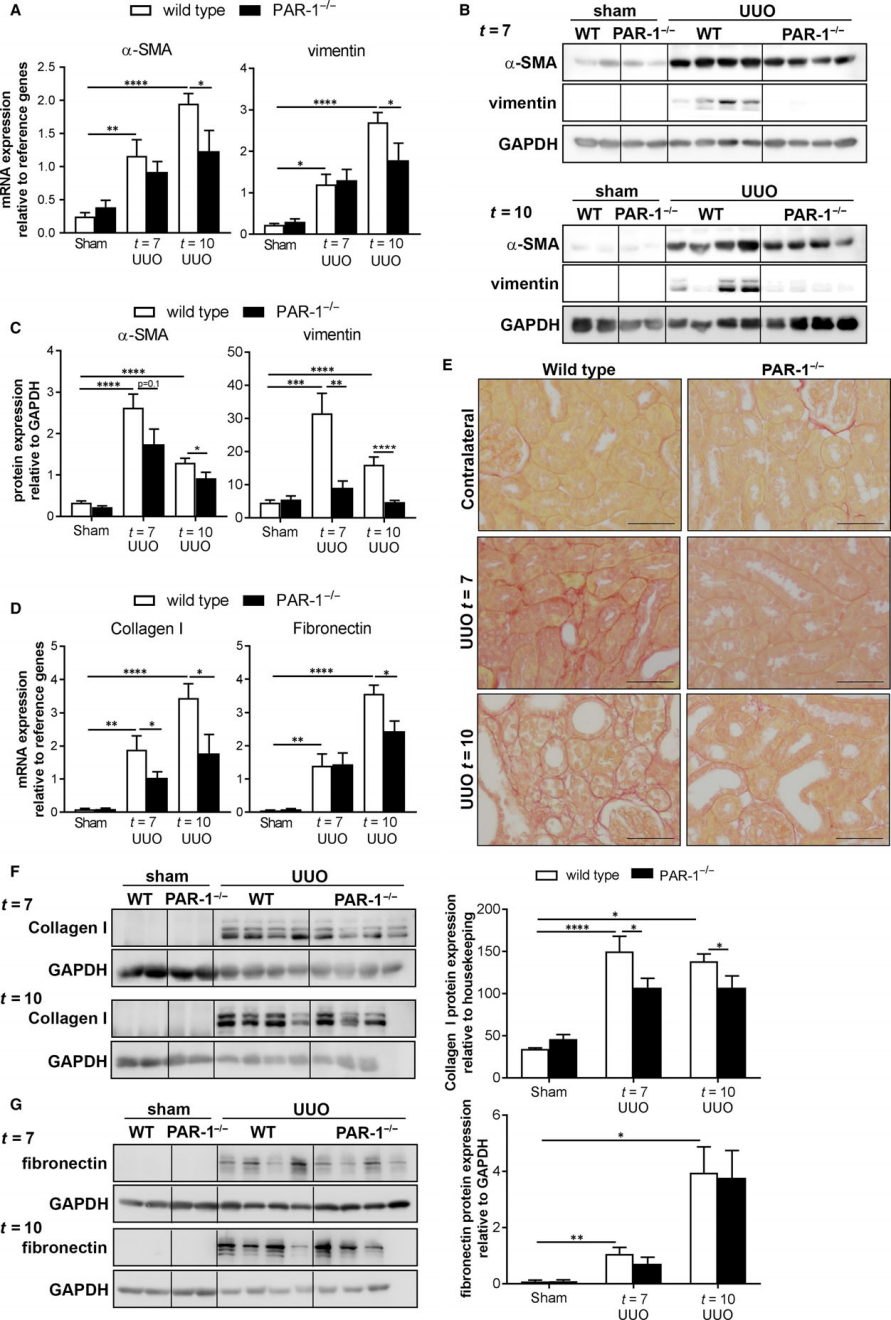
PAR‐1 deficiency limits renal fibrosis. A, α‐SMA and vimentin mRNA expression in whole kidney lysates of unobstructed (sham) and obstructed (UUO) kidneys of wild‐type (WT) and PAR‐1‐deficient (PAR‐1^−/−^) mice, 7 and 10 d after UUO. B, Western blot analysis of α‐SMA and vimentin in whole kidney lysates of unobstructed (sham) and obstructed (UUO) kidneys of wild‐type (WT) and PAR‐1‐deficient (PAR‐1^−/−^) mice, 7 and 10 d after UUO. GAPDH expression served as loading control. C, Quantification of Western blots depicted in panel B. D, mRNA expression of collagen I and fibronectin in whole kidney lysates of unobstructed (sham) and obstructed (UUO) kidneys of wild‐type (WT) and PAR‐1‐deficient (PAR‐1^−/−^) mice, 7 and 10 d after UUO. E, Representative pictures of picrosirius red staining. F‐G, Western blot analysis (left: representative picture, right: quantification) of collagen I (F) and fibronectin (G) in whole kidney lysates of unobstructed (sham) and obstructed (UUO) kidneys of wild‐type (WT) and PAR‐1‐deficient (PAR‐1^−/−^) mice, 7 and 10 d after UUO. GAPDH expression served as loading control. *P < 0.05; **P < 0.01; ***P < 0.005; ****P < 0.001 (one‐way ANOVA followed by Bonferroni multiple comparisons test)

To assess whether the diminished renal injury in PAR‐1‐deficient mice translated into a diminished fibrotic response, we next analysed fibroblast deposition by assessing expression levels of the fibroblast markers α‐SMA and vimentin and extracellular matrix molecules collagen I and fibronectin. As shown in Figure 3A‐C, UUO‐induced α‐SMA and vimentin levels were significantly higher in wild‐type mice as in PAR‐1‐deficient mice both on the mRNA (Figure 3A) and protein (Figure 3B, C) level. Diminished fibroblast accumulation in PAR‐1‐deficient mice was accompanied by diminished collagen deposition (Figure 3D‐F). Indeed, UUO‐induced collagen mRNA levels were lower in PAR‐1‐deficient mice as compared to wild‐type mice (Figure 3D). To confirm the difference on the protein level, we next performed picrosirius red stainings to visualize collagen fibres in obstructed kidneys and western blots to actually quantitative collagen levels in kidney homogenates of wild‐type and PAR‐1‐deficient mice. As shown in Figure 3E, picrosirius red positive collagen fibres are omnipresent in obstructed kidneys of wild‐type mice and seem diminished in obstructed kidneys of PAR‐1‐deficient mice. Western blot analysis, as depicted in Figure 3F, confirms that collagen I was significantly reduced in PAR‐1‐deficient mice. Finally, we show that diminished collagen deposition was not accompanied by reduced fibronectin production. Although fibronectin mRNA levels were reduced in PAR‐1‐deficient mice compared to wild‐type mice at t = 10, western blot analysis showed that protein levels did not differ between wild type and PAR‐1‐deficient mice (Figure 3G). Overall, these data show that PAR‐1 deficiency limits UUO‐induced fibroblast accumulation with subsequent collagen deposition.

### 3.3 No evidence that PAR‐1 deficiency preserves the epithelial phenotype of tubular epithelial cells after UUO

**Figure 4:**
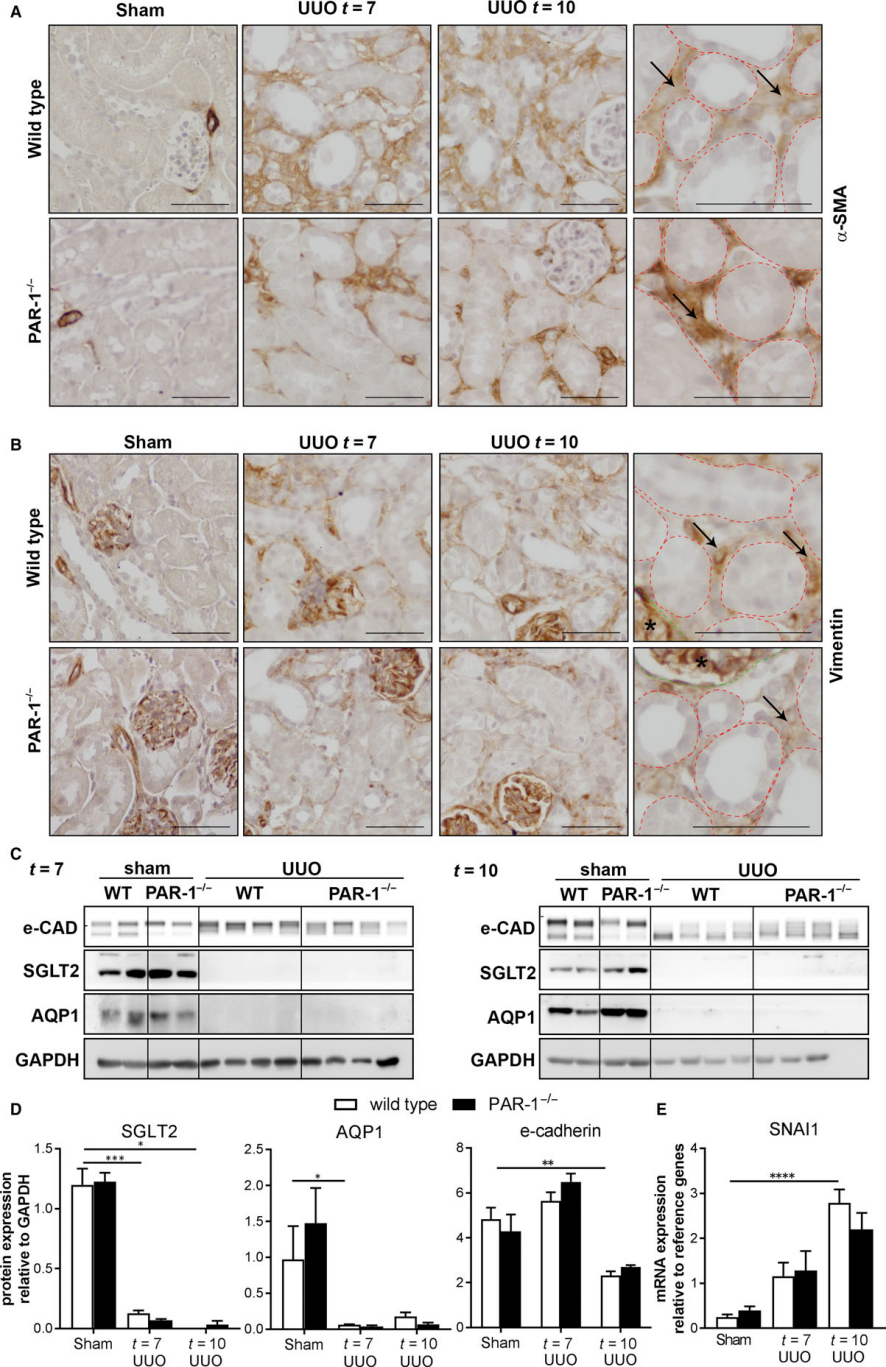
Interstitial expression of mesenchymal markers, in wild‐type and PAR‐1‐deficient mice. Representative images of α‐SMA (A)‐ and vimentin (B)‐stained kidney slides of wild‐type and PAR‐1‐deficient mice 7 and 10 d after UUO and in unobstructed (Sham) control kidneys; scale bars represent 50 μm. C, Western blot analysis of E‐cadherin, SGLT2 and AQP1 in whole kidney lysates of unobstructed (sham) and obstructed (UUO) kidneys of wild‐type (WT) and PAR‐1‐deficient (PAR‐1^−/−^) mice, 7 and 10 d after UUO. GAPDH expression served as loading control. D, Quantification of Western blots depicted in panel C. E, SNAI1 mRNA expression in whole kidney lysates of unobstructed (sham) and obstructed (UUO) kidneys of wild‐type (WT) and PAR‐1‐deficient (PAR‐1^−/−^) mice, 7 and 10 d after UUO. *P < 0.05; **P < 0.01; ***P < 0.005; ****P < 0.001 (one‐way ANOVA followed by Bonferroni multiple comparisons test)

It is tempting to speculate that the observed differences in vimentin and α‐SMA levels between wild‐type and PAR‐1‐deficient mice (Figure 3A, B) suggest that PAR‐1 also drives EMT after the induction of UUO. Indeed, vimentin and α‐SMA are both well‐known markers of EMT, but vimentin and α‐SMA expression may also originate from infiltrating/proliferating fibroblasts rather than from transitioned TECs.^24^, ^25^ To discriminate between these processes, immunohistochemical analysis of α‐SMA and vimentin was performed to localize the α‐SMA and vimentin expressing cells. Interestingly, both these markers were mainly expressed in the renal interstitium and hardly any positive TEC was found (Figure 4A, B) suggesting that the observed reduction of vimentin and α‐SMA more likely reflects diminished interstitial fibroblast accumulation, rather than reduced EMT of TECs. Moreover, no difference in protein expression levels of the epithelial markers e‐cadherin, SGLT2, and AQP‐1 was observed between wild‐type and PAR‐1‐deficient mice (Figure 4C, D). Finally, we observed that SNAI1 expression (a transcription factor specific for EMT) was significantly induced at day 10 after the induction of UUO but no difference between wild‐type and PAR‐1‐deficient mice was found (Figure 4E). Overall, we only observe minimal signs of EMT in both wild‐type and PAR‐1‐deficient mice suggesting PAR‐1‐dependent EMT is of minor importance in UUO‐induced pathology.

### 3.4 PAR‐1 stimulation leads to MCP‐1 and TGF‐β secretion

**Figure 5:**
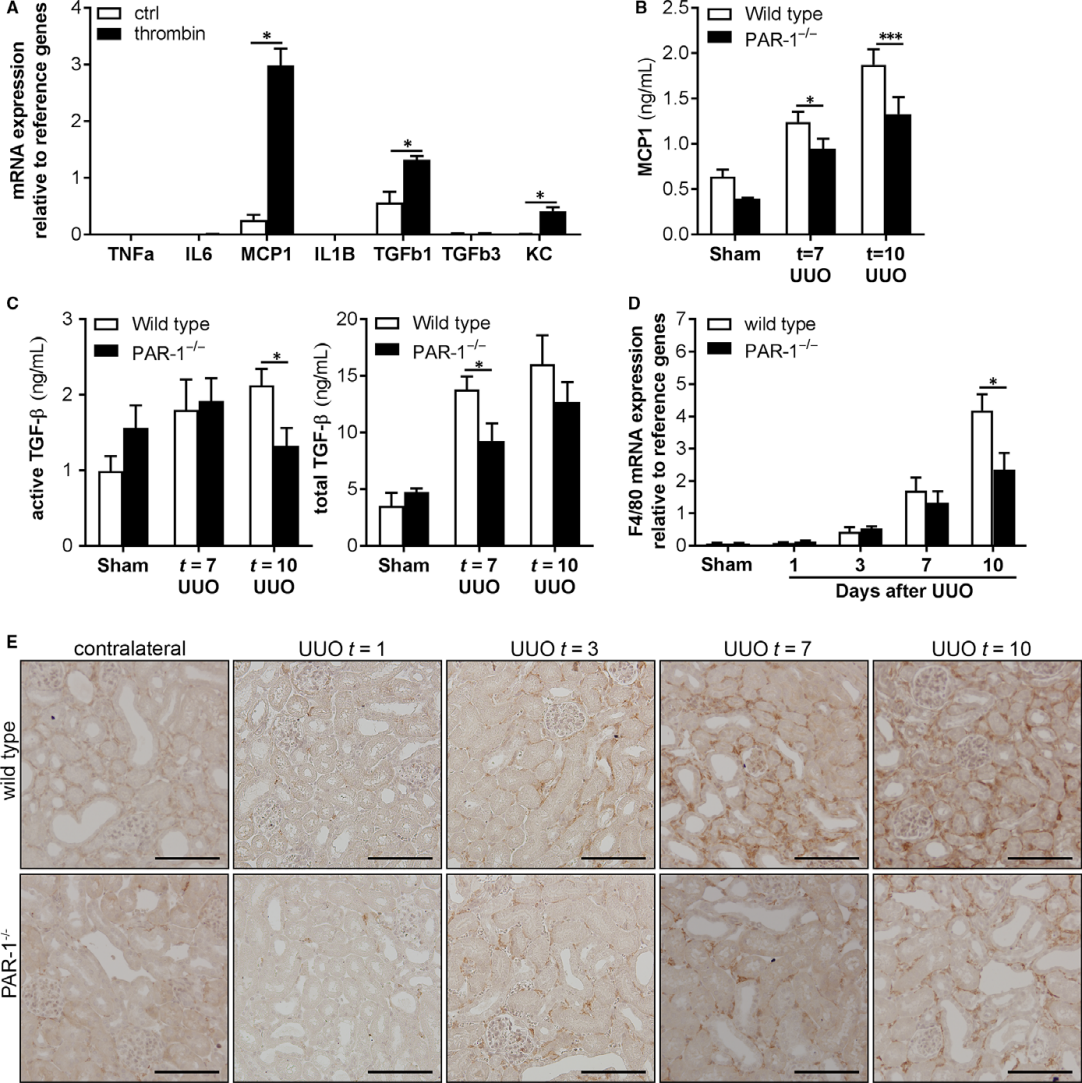
PAR‐1 activation induces pro‐fibrotic cytokine expression and potentiates macrophage influx during renal fibrosis. A, imPTEC mRNA expression of cytokines 24 h after PAR‐1 stimulation with thrombin (1 U/mL). Indicated is the average of three independent experiments. B‐C, Protein expression of MCP‐1 (B), total TGF‐β and active TGF‐β (C) measured by ELISA in whole kidney lysates of wild‐type and PAR‐1‐deficient (PAR‐1^−/−^) mice 7 and 10 d after UUO and in unobstructed control kidneys. D, F4/80 (ie, macrophage marker) mRNA expression in kidney lysates of wild‐type and PAR‐1‐deficient (PAR‐1^−/−^) mice 1, 3, 7, and 10 d after UUO and in unobstructed (Sham) control kidneys. E: Representative images of F4/80‐stained kidney slides of wild‐type and PAR‐1‐deficient (PAR‐1^−/−^) mice 1, 3, 7, and 10 d after UUO and in unobstructed (Sham) control kidneys; scale bars represent 100 μm. *P < 0.05; ***P < 0.005; ****(*t* test [A] and one‐way ANOVA followed by Bonferroni multiple comparisons test [B‐D])

Next to its role in EMT, PAR‐1 has also been implicated in the production of pro‐fibrotic and/or pro‐inflammatory mediators, like MCP‐1 and TGF‐β, during pulmonary fibrosis.^13^, ^26^ To determine whether PAR‐1 would play a role in the production of these pro‐fibrotic and/or pro‐inflammatory mediators in the setting of renal fibrosis as well, we assessed PAR‐1‐induced cytokine production by TECs in vitro. As shown in Figure 5A, thrombin‐dependent PAR‐1 activation induced expression levels of MCP‐1, TGF‐β1, and KC, but not of TNFα, IL6, or TGF‐β3 in TECs. In line with these in vitro data, MCP‐1 and TGF‐β levels were also reduced in obstructed kidneys of PAR‐1‐deficient mice compared to obstructed kidneys of wild‐type mice (Figure 5B, C). Importantly, reduced MCP‐1 levels in PAR‐1 deficient obstructed kidneys were accompanied by a reduced influx of macrophages (shown as representative pictures in Figure 5E, and quantified by F4/80 expression as shown in Figure 5D). Together, these data show that PAR‐1 induces the expression of pro‐fibrotic mediators with subsequent macrophage influx thereby providing an alternative explanation for the observed reduced renal fibrosis in PAR‐1‐deficient mice.

## 4 DISCUSSION

Renal fibrosis is a life‐threatening complication with limited treatment options and novel treatment options are thus eagerly awaited for. As EMT is postulated to contribute to the development of renal fibrosis^27^, we aimed to elucidate the relevance of PAR‐1, a proposed mediator of EMT, during renal fibrosis. We show that PAR‐1 activation induces EMT of proximal TECs in vitro and that PAR‐1 deficiency limits renal fibrosis after experimental UUO. Diminished fibrosis in PAR‐1‐deficient mice is, however, not associated with reduced EMT but actually associates with diminished fibroblast accumulation and reduced pro‐fibrotic cytokine production and macrophage recruitment.

To elucidate the underlying mechanism by which PAR‐1 would limit UUO‐induced renal fibrosis, we hypothesized that PAR‐1 activation drives EMT thereby promoting renal fibrosis. In vitro, PAR‐1 activation‐induced differentiation of TECs into α‐SMA and vimentin positive mesenchymal cells expressing collagen I and fibronectin while losing epithelial gene expression. In vivo, however, we did not observe a difference in UUO‐induced EMT between wild‐type and PAR‐1‐deficient mice. Although mesenchymal marker expression (ie, vimentin and α‐SMA) was significantly reduced in PAR‐1‐deficient mice subjected to UUO, this was mainly due to reduced expression in the interstitium and hardly any positive α‐SMA or vimentin positive TECs were identified in both wild‐type and PAR‐1‐deficient mice. Moreover, expression levels of epithelial markers E‐cadherin, AQP‐1, and SGLT2 decreased significantly after the induction of UUO but the decrease was similar in wild‐type and PAR‐1 deficient mice. Importantly, although decreased expression levels of epithelial markers are considered signs representative of EMT, the decrease may well represent epithelial damage instead. Finally, expression levels of the key EMT transcription factor SNAI1 were also similar between wild‐type and PAR‐1‐deficient mice. Overall, we thus did not obtain any evidence that PAR‐1 deficiency preserves the epithelial phenotype of tubular epithelial cells in vivo. It is important to stress, however, that EMT is difficult to assess in vivo using (epithelial and/or mesenchymal) marker expression and actually may only be quantitatively assessed using cell fade tracing studies.^25^, ^28^

Despite the lack of effect of PAR‐1 on EMT observed in vivo, fibroblast accumulation and collagen deposition were clearly diminished in PAR‐1‐deficient mice, suggesting that mechanisms other than EMT are involved in PAR‐1‐dependent renal fibrosis. Indeed, tracing studies show that the interstitial accumulation of myofibroblasts during renal fibrosis arises mainly from proliferating resident fibroblasts and infiltration of bone marrow derived fibroblasts, instead of originating from the transitioned TECs. In fact, the amount of interstitial myofibroblasts that originate from epithelial cells was estimated at about 5% only.^25^ Nevertheless, disabling EMT by conditional knockout of Snail or Twist specifically in tubular epithelial cells revealed that EMT is essential for the development of renal fibrosis. The mechanisms by which activated TECs contribute to fibrosis other than a full transition towards myofibroblasts remain elusive. Potential alternatives include partial EMT leading to impaired proliferation and diminished regenerative capacity, or secretome changes resulting in stimulation of interstitial fibroblasts.^27^ EMT of single TECs may already result in secretome changes sufficient to induce fibroblast accumulation. Small PAR‐1‐dependent changes in EMT, which do not change overall AQP‐1 and ZO‐1 levels, and which are not easily detected immunohistochemically, may consequently affect fibroblast accumulation and subsequent renal fibrosis. Irrespective of the actual role of EMT in renal fibrosis, our results identify PAR‐1 as a novel mediator of renal fibrosis.

Interestingly, PAR‐1 deficiency led to a marked decrease of interstitial macrophage accumulation upon UUO providing an alternative explanation on how PAR‐1 potentiates renal fibrosis. As we show that TECs secrete MCP‐1 after PAR‐1 stimulation, we postulate that PAR‐1‐dependent macrophage recruitment, which has been observed before in pulmonary fibrosis,^26^, ^29^ is likely due to TEC‐dependent MCP‐1 expression. In addition, PAR‐1 may also directly potentiate the intrinsic migratory activity of macrophages.^26^, ^29^ Once recruited, macrophages secrete large amounts of pro‐fibrotic cytokines like TGF‐β, which in turn induce fibroblast proliferation.^7^, ^30^ It is thus tempting to speculate that reduced macrophage recruitment in PAR‐1‐deficient mice results in the observed reduction in fibroblast accumulation with subsequent reduced extracellular matrix production and renal fibrosis. Additionally, PAR‐1‐dependent TGF‐β production by TECs, as observed in our in vitro experiments and as described before for HK2 cells^31^, may further induce fibroblast proliferation and activation thereby further enhancing renal fibrosis. This latter notion is supported by recent findings that PAR‐1 stimulation of cardiac fibroblasts also leads to TGF‐β production with subsequent myofibroblast accumulation.^32^

Although PAR‐1 has originally been identified as blood coagulation factor receptor, at least 12 different proteases have already been described to activate PAR‐1 in different pathological settings (REF). The identification of the endogenous PAR‐1 agonist in the setting of UUO‐induced renal fibrosis is therefore a major challenge. Interestingly, however, during preparation of our manuscript, it was shown that UUO‐induced renal damage and tubulointerstitial fibrosis was suppressed in UUO mice treated with the specific FXa inhibitor edoxaban.^23^ Although this may pinpoint FXa as the endogenous PAR‐1 agonist driving PAR‐1‐dependent renal injury during UUO, it was not shown that the effect of FX inhibition was PAR‐1 dependent.

Overall, we here show that PAR‐1 contributes to renal fibrosis, as evident from increased fibroblast accumulation and collagen deposition in the interstitial areas of wild‐type kidneys compared to PAR‐1‐deficient kidneys, and could therefore be a potential target to pursue in the setting of renal fibrosis. Based on both in vivo and in vitro results we propose that PAR‐1 potentiates renal fibrosis by regulating the expression of pro‐fibrotic mediators MCP‐1 and TGF‐β subsequently leading to MCP‐1‐induced macrophage influx and TGF‐β‐induced extracellular matrix production. Subsequent experiments addressing pharmacological inhibition of PAR‐1 should elucidate whether PAR‐1 inhibition indeed has clinical potential for renal fibrosis.

## CONFLICT OF INTEREST

All authors declare no conflict of interest.

## AUTHOR CONTRIBUTIONS

MW designed experiments, researched data, and wrote the manuscript. DMR designed experiments, researched data and reviewed the manuscript. SF contributed to conception, discussion and reviewed the manuscript. JWD contributed to conception, design of experiments and discussion and reviewed the manuscript. CAS contributed to conception, design of experiments and discussion and reviewed the manuscript.
